# Significantly Increased Visceral Adiposity Index in Prehypertension

**DOI:** 10.1371/journal.pone.0123414

**Published:** 2015-04-10

**Authors:** Yanan Ding, Dongfeng Gu, Yanxuan Zhang, Wenjie Han, Hengliang Liu, Qingshan Qu

**Affiliations:** 1 Division of Cardiovascular Medicine, The People’s Hospital of Zhengzhou, Affiliated with Southern Medical University, Zhengzhou, P.R. China; 2 Department of Nephrology and Transplantation Center, The People’s Hospital of Zhengzhou, Affiliated with Southern Medical University, Zhengzhou, P.R. China; Faculty of Biology, SPAIN

## Abstract

**Background:**

The prevalence of prehypertension has increased in China, and prehypertension frequently progress to hypertension over a short time period; both have become public health problems. Therefore, this study was conducted to determine the relationship between the Visceral Adiposity Index (VAI) and blood pressure (BP) in China.

**Methods:**

A cross-sectional epidemiological survey was conducted in China using a stratified random cluster sampling method. Sex-specific VAI quartile cut-off points were used as follows: 0.88, 1.41, 2.45 in males and 0.85, 1.33, 2.22 in females. Prehypertension and hypertension were each defined according to The Seventh Report of the Joint National Committee on the Prevention, Detection, Evaluation, and Treatment of High Blood Pressure (JNC 7) guidelines. A multivariate logistic analysis was conducted to analyze the relationship among VAI, prehypertension and hypertension.

**Results:**

The ORs for prehypertension and hypertension in the upper quartiles of the VAI were 1.514 (1.074-2.133), P=0.018 and 1.660 (1.084-2.542), P=0.020, in males, after adjusting for age, education, smoking habits, alcohol consumption, physical activity, serum creatinine, fasting glucose, and plasma insulin. Following further adjustments for the above confounders, chronic kidney disease, and diabetes, the ORs for prehypertension and hypertension in the upper quartile of the VAI were 1.660 1.533 (1.086-2.165), P=0.015, and 1.743 (1.133-2.680), P=0.011, in males. The ORs for prehypertension and hypertension in the upper quartile of the VAI were 1.691 (1.223-2.338), P=0.001, and 1.682 (1.162-2.435), P=0.006, in females, after adjusting for age, education, smoking habits, alcohol consumption, physical activity, serum creatinine, fasting glucose, and plasma insulin. Following further adjustments for the above confounders, chronic kidney disease, and diabetes, the ORs for prehypertension and hypertension in the upper quartile of the VAI were 1.688 (1.220-2.334), P=0.002, and 1.657 (1.141-2.406), P=0.008, in females.

**Conclusions:**

A higher VAI was positively associated with both prehypertension and hypertension in both males and females. It is both essential and urgent that clinicians take steps to control and prevent visceral adiposity.

## Introduction

Many studies have shown that visceral fat dysfunction has a close relationship with cardiovascular disease as a result of increased adipokine production, proinflammatory activity, and decreased insulin sensitivity [1–3]. Waist circumference (WC) has been used to assess visceral adiposity; however, this parameter alone does not help in distinguishing between subcutaneous and visceral fat masses [4]. Therefore, the Visceral Adiposity Index (VAI), which is based on WC, body mass index (BMI), triacylglycerols (TG), and high density lipoprotein cholesterol (HDL-C) and was recently introduced by the AlkaMeSy Study Group [5], was used as a marker of both visceral fat dysfunction and an individual’s subsequent cardiometabolic risk.

Over the past several decades, various studies have indicated that hypertension represents a major health threat, as affected individuals carry an increased risk of cardiovascular disease, stroke, renal failure, and vision loss [6–10], and hypertension has become an important public-health problem worldwide [11]. Prehypertension frequently progresses to hypertension within a period of 4 years, particularly in older adults, as observed in an American population by the Framingham Heart Study [12], and in a Western European population by the Flemish Study on Environment, Genes and Health Outcomes [13]. Prehypertension is also associated with an increased risk of cardiovascular disease, with a risk-factor-adjusted hazard ratio of 2.5 in women and 1.6 in men, compared with individuals with optimal blood pressures [14].

However, only limited numbers of studies have examined the relationship between VAI and blood pressure (BP), therefore, we conducted an epidemiological survey to determine the relationship among VAI, prehypertension and hypertension in China.

## Methods

### Ethics Statement

Each of the participants provided written informed consent prior to data collection. Illiterate participants were read information about the study and provided a thumb impression. The Human Ethics Committee of The People’s Hospital of Zhengzhou, Affiliated with Southern Medical University, Zhengzhou, China, approved the study.

### Participants

A community-based survey was conducted in Zhengzhou from October 2011 to October 2012 to investigate the prevalence of prehypertension. Participants were selected using a stratified random cluster sampling method. Three districts were first selected randomly; three additional communities were subsequently selected randomly from each district. Finally, each of the residents who had lived in Zhengzhou for at least 5 years and was a member of one of the chosen communities was selected and invited to participate our survey. A total of 4065 participants were selected from a pool of 4800 citizens who completed the entire survey, a response rate of 84.6%.

### Questionnaire

All clinical doctors, technicians, medical students and nurses who participated in the project received intensive training regarding proper screening methods. All participants were asked to complete the questionnaire under the guidance of a well-trained investigator. The questionnaire consisted of questions regarding age, sex, a personal history of diabetes (yes vs. no), a personal history of hypertension (yes vs. no), a personal history of cardiovascular disease (yes vs. no), education (>10 years vs. 6–10 years vs. 1–5 years vs. no), >10 years equal to above high school in China, smoking habits (yes[current] vs. yes[former] vs. no), alcohol intake (yes[current] vs. yes[former] vs. no), and physical activity (>60 min/day vs. 30–60 min/day vs. <30 min/day vs. no), >60 min/day and 30–60 min/day were considered as active physical activity, and <30 min/day and no were considered as inactive physical activity; WC, height and BP were each measured manually. Prior to BP measurements, participants were seated quietly for 5 to 10 minutes in a chair with arm supported at heart level and the rotator cuff positioned 3 cm above the antecubital fossa, BP was measured using Omron (SEM 1 Model) automatic BP monitor (Omron Healthcare Co., Ltd., IL, USA) with an appropriate cuff size, and the same cuff was used for a participant’s subsequent visits [15, 16]. Study visits were approximately at the same time of day. Average BP was then calculated from three measurements. BMIs were calculated using the following equation: BMI = weight (kg)/height (m^2^). Previously diagnosed disease was determined based on a positive answer to the following question: “Has a doctor ever told you that you have hypertension?” Two-level variants of education status (above high school vs. below high school), physical activity (active physical activity vs. inactive physical activity), smoking (current smoking vs. non current smoking) and alcohol (current alcohol vs. non current alcohol) were analyzed.

### Blood and urine sample collection

Appointments were scheduled for both urine and blood collection. Participants were asked to provide a sample of their first morning urine, as well as a midstream urine sample; no protease inhibitor was used. Menstrual periods were avoided among female participants. Fasting venous blood draws were performed either at local community clinics or at health stations. All urine and blood samples were sent to the central laboratory of The People’s Hospital of Zhengzhou, Affiliated with Southern Medical University. The blood and urine samples were either disposed of within 3 hours or stored at 4°C for as long as two days. The central laboratory successfully completed a standardization and certification program.

### Blood and urine measurements

FPG (fasting plasma glucose, FPG) testing was performed, and fasting plasma insulin concentrations were measured via an electrochemical luminescence immunoassay. Serum total cholesterol (TC), high-density lipoprotein cholesterol (HDLC), triacylglycerols (TG), and low-density lipoprotein cholesterol (LDLC) were each measured using an autoanalyzer (Toshiba, Japan). Serum creatinine (Scr) was measured using overnight fasting venous blood samples, via Jaffe’s kinetic method. Albuminuria was measured using immunoturbidimetric tests.

### Evaluation criteria

VAI, a sex-specific index based on WC, BMI, TG and HDLC, was calculated as follows [5]:

$$\begin{array}{l} \text{Males: VAI = }\left(\frac{\text{WC}}{\text{39.68+}\left(\text{1.88×}BMI\right)}\right)\text{ × }\left(\frac{\text{TG}}{\text{1.03}}\right)\text{ × }\left(\frac{\text{1.31}}{\text{HDL}}\right)\\ \text{Females: VAI = }\left(\frac{\text{WC}}{\text{39.58+}\left(\text{1.89×BMI}\right)}\right)\text{ × }\left(\frac{\text{TG}}{0.81}\right)\text{ × }\left(\frac{\text{1.52}}{\text{HDL}}\right) \end{array}$$

The classifications of normotension, prehypertension and hypertension were each based on the classifications of BP as determined by the JNC-7 [17]. Normotension was defined as not requiring antihypertensive medication, and as having a systolic blood pressure (SBP) < 120 mm Hg and a diastolic blood pressure (DBP) <80 mm Hg. Prehypertension was defined as not requiring antihypertensive medication, and as having an SBP of 120–139 mm Hg or a DBP of 80–89 mm Hg. Hypertension was defined as an SBP ≥ 140 mm Hg or a DBP ≥ 90 mm Hg, as well as the requirement of an antihypertensive medication. A family history of hypertension was defined as the diagnosis of hypertension in at least one parent.

The urinary albumin to creatinine ratio (ACR; mg/g creatinine) was calculated, and an ACR greater than 30 mg/g was defined as albuminuria. An eGFR (estimated glomerular filtration rate, eGFR) was calculated according to the equation developed by the Modification of Diet in Renal Disease (MDRD) Study [18, 19]. Reduced renal function was defined as an eGFR less than 60 ml/min/1.73 m^2^. Chronic kidney disease (CKD) was defined as either reduced renal function or as albuminuria, according to KIDIGO [20]. The diagnosis of diabetes mellitus (DM) was based on the following criteria developed by the American Diabetes Association: an A1C ≥ 6.5%, an FPG ≥ 126 mg/dL or a 2-h plasma glucose level ≥ 200 mg/dL during an OGTT [21].

### Statistical analysis

Acquired data were analyzed using SPSS 13.0 (SPSS Inc., Chicago, IL, USA). Continuous variables were represented as means ± SDs, and categorical variables were represented as proportions of each subgroup.

Each of the participants was placed into one of the following two groups: men and women; each participant was subsequently placed into one of the following three subgroups: normotension, prehypertension and hypertension. The basic characteristics of the four VAI quartiles were examined in both the male and the female groups. Continuous variables were analyzed via one-way ANOVA, and categorical variables were analyzed via the Chi-square test or the Fisher’s exact test.

Logistic regression models were used to determine whether VAI is associated with both prehypertension and hypertension in both men and women. VAI was divided into four quartiles and considered a categorical variable. Model one was adjusted for lifestyle (smoking status, alcohol consumption, physical activity, and education), VAI, serum creatinine, fasting glucose, and plasma insulin. To determine whether CKD and DM affect the relationship between VAI and BP, each parameter was included in model two. The lower quartile was used as a reference category. Logistic regression was performed separately in men and women. P values less than 0.05 were considered statistically significant.

## Results

Four-thousand-sixty-five participants completed the survey, and 115 participants were excluded due to either missing blood glucose data or missing anthropometric data. The 1688 men (42.7%) and 2262 women (57.3%) were of Han Ethnic. The mean age of the subjects who participated in this study was 44.8 ± 13.0 years.

### The basic characteristics of the male and female participants

As shown in Table 1, there were significant differences in smoking habits, alcohol consumption, physical activity, WC, BMI, DBP, serum glucose, plasma insulin, TG, HDLC, TC, and LDLC in males, as the upper VAI quartile participants exhibited higher DBP, serum glucose, plasma insulin, TG, TC, and LDLC, and lower HDLC, prevalence of smoking, and prevalence of alcohol consumption, compared with the lower VAI subjects, among the males enrolled in this study.

**Table 1:** The basic characteristics of the male participants.

	VAI (male)				*P*
	1^st^ Quartile (n = 422)	2^nd^ Quartile (n = 422)	3^rd^ Quartile (n = 422)	4^th^ Quartile (n = 422)	
Age (years)	42.8±14.9	43.6±13.7	44.3±13.0	44.3±11.9	0.329
Education status (≥high school) (%)	176(41.7)	153(36.3)	138(32.7)	163(38.6)	0.050
Current Smoking (%)	277(65.6)	264(62.6)	242(57.3)	216(51.2)	*P*<0.001
Current Alcohol (%)	285(67.5)	268(63.5)	247(58.5)	215(50.9)	*P*<0.001
Inactive Physical Activity (%)	134(31.8)	143(33.9)	157(37.2)	174(41.2)	*P*<0.05
Prehypertension (%)	143(33.9)	147(34.8)	146(34.6)	175(41.5)	0.076
Hypertension (%)	89(21.1)	113(26.8)	100(23.7)	118(28.0)	0.089
Chronic Kidney Disease (%)	58(13.7)	56(13.3)	62(14.7)	64(15.2)	0.855
Diabetes Mellitus (%)	20(4.7)	18(4.3)	18(4.3)	27(6.4)	0.428
Waist Circumference (cm)	80.28±8.98	84.31±8.38	86.26±7.43	89.62±7.77	*P*<0.001
Body Mass Index (kg/m^2^)	23.93±2.89	23.62±3.42	23.79±3.01	24.82±2.86	*P*<0.001
Systolic Blood Pressure (mmHg)	121±19	122±19	122±18	123±17	0.167
Diastolic Blood Pressure (mmHg)	74±11	76±11	76±10	78±10	*P*<0.001
Serum Creatinine (μmol/L)	80.9±48.8	78.7±23.2	78.2±19.4	83.3±76.7	0.386
Serum glucose (mmol/L)	4.9±1.0	5.0±1.1	5.0±1.2	5.4±1.9	*P*<0.001
Plasma insulin (μU/mL)	5.0±5.0	5.8±5.5	6.1±5.3	7.6±6.3	*P*<0.001
Total Cholesterol (mmol/L)	4.5±0.9	4.8±0.9	4.9±1.0	5.3±1.1	*P*<0.001
High-Density Lipoprotein Cholesterol (mmol/L)	1.7±0.5	1.4±0.3	1.2±0.3	1.1±0.3	*P*<0.001
Triacylglycerols (mmol/L)	0.8±0.3	1.2±0.3	1.7±0.5	3.7±2.2	*P*<0.001
Low-Density Lipoprotein Cholesterol (mmol/L)	2.4±0.8	2.8±0.9	2.9±0.8	2.5±1.1	*P*<0.001

Means ± SDs represented the continuous variables, and proportions represented the categorical variables.

1^st^ quartile of VAI: 0–0.88; 2^nd^ quartile of VAI: 0.89–1.41; 3^rd^ quartile of VAI: 1.42–2.45; 4^th^ quartile of VAI: ≥2.46.

Continuous variables were analyzed via One-way ANOVA, categorical variables were analyzed via the Chi-square test or Fisher’s exact test, and P value less than 0.05 was considered statistical significant.

As shown in Table 2, there were significant differences in age, education, physical activity, prehypertension, hypertension, DM, WC, BMI, SDP, DBP, Scr, serum glucose, plasma insulin, TG, HDLC, TC, and LDLC in females; the upper VAI quartile participants exhibited higher ages, prevalence of prehypertension, prevalence of hypertension, DM, SBP, DBP, Scr, serum glucose, plasma insulin, TG, TC, and LDLC, and lower HDLC and physical activity levels, compared with the lower VAI quartile subjects, among females.

**Table 2:** The basic characteristics of the female participants.

	VAI (female)				*P*
	1^st^ Quartile (n = 565)	2^nd^ Quartile (n = 566)	3^rd^ Quartile (n = 566)	4^th^ Quartile (n = 565)	
Age (years)	40.8±11.8	43.2±12.5	48.0±12.3	50.7±11.5	*P*<0.001
Education status (≥high school) (%)	278(49.2)	289(51.1)	362(64.0)	388(68.7)	*P*<0.001
Current Smoking (%)	71(12.6)	73(12.9)	76(13.4)	80(14.2)	0.870
Current Alcohol (%)	64(11.3)	67(11.8)	72(12.7)	83(14.7)	0.339
Inactive Physical Activity (%)	209(37.0)	190(33.6)	166(29.3)	154(27.3)	*P*<0.05
Prehypertension (%)	113(20.0)	144(25.4)	167(29.5)	168(29.7)	*P*<0.001
Hypertension (%)	80(14.2)	99(17.5)	148(26.1)	203(35.9)	*P*<0.05
Chronic Kidney Disease (%)	93(16.5)	89(15.7)	86(15.2)	84(14.9)	0.891
Diabetes Mellitus (%)	20(3.5)	33(5.8)	39(6.9)	84(14.9)	*P*<0.001
Waist Circumference (cm)	73.1±8.5	76.0±8.93	78.4±8.9	82.8±8.6	*P*<0.001
Boby Mass Index (kg/m^2^)	21.4±2.7	22.3±3.1	23.0±3.1	24.4±3.1	*P*<0.001
Systolic Blood Pressure (mmHg)	112±18	116±19	122±22	126±21	*P*<0.001
Diastolic Blood Pressure (mmHg)	70±9.6	72.4±10	75±11	78±12	*P*<0.001
Serum Creatinine (μmol/L)	67.4±21.1	69.3±46.8	73.4±37.3	79.8±63.6	*P*<0.001
Serum glucose (mmol/L)	4.7±0.8	4.9±0.9	5.1±1.4	5.5±1.7	*P*<0.001
Plasma insulin (μU/mL)	5.7±4.7	6.9±5.2	8.2±5.5	9.8±6.4	*P*<0.001
Total Cholesterol (mmol/L)	4.6±1.0	4.7±1.0	5.1±1.1	5.4±1.2	*P*<0.001
High-Density Lipoprotein Cholesterol (mmol/L)	1.9±0.7	1.6±0.5	1.5±0.4	1.3±0.4	*P*<0.001
Triacylglycerols (mmol/L)	0.6±0.2	1.0±0.3	1.3±0.4	2.6±1.5	*P*<0.001
Low-Density Lipoprotein Cholesterol (mmol/L)	2.3±0.9	2.5±0.8	2.8±0.8	2.8±1.0	*P*<0.001

Means ± SDs represented the continuous variables, and proportions represented the categorical variables.

1^st^ quartile of VAI: 0–0.85; 2^nd^ quartile of VAI: 0.86–1.33; 3^rd^ quartile of VAI: 1.34–2.22; 4^th^ quartile of VAI: ≥2.23.

Continuous variables were analyzed via One-way ANOVA, categorical variables were analyzed via the Chi-square test or Fisher’s exact test, and P value less than 0.05 was considered statistical significant.

### The relationship between VAI and blood pressure in males and females

As shown in Table 3, the VAI was significantly associated with both prehypertension and hypertension in males, after adjusting for age, education, smoking habits, alcohol consumption, physical activity, serum creatinine, fasting glucose, and plasma insulin; the ORs for prehypertension and hypertension in the upper quartile of the VAI were 1.514 (95%CI 1.074–2.133), P = 0.018, and 1.533 (95%CI 1.086–2.165), P = 0.015, in males. Following further adjustments for the above confounders, CKD, and DM, the ORs for prehypertension and hypertension in the upper quartile of the VAI were 1.660 (95CI 1.084–2.542), P = 0.020 and 1.743 (95CI 1.133–2.680), P = 0.011, in males.

**Table 3:** The relationship between visceral adiposity index and blood pressure levels in males.

VAI	Prehypertension (Male)				Hypertension (Male)			
	Model one ^a^		Model two ^b^		Model one ^a^		Model two ^b^	
	OR(95% CI)	*P*	OR(95% CI)	*P*	OR(95% CI)	*P*	OR(95% CI)	*P*
1^st^ Quartile	Reference		Reference		Reference		Reference	
2^nd^ Quartile	1.130(0.815–1.568)	0.464	1.134(0.816–1.576)	0.453	1.046(0.688–1.589)	0.835	1.069(0.701–1.630)	0.758
3^rd^ Quartile	0.964(0.695–1.337)	0.825	0.975(0.702–1.353)	0.878	1.489(0.983–2.256)	0.060	1.542(1.015–2.343)	0.042
4^th^ Quartile	1.514(1.074–2.133)	0.018	1.533(1.086–2.165)	0.015	1.660(1.084–2.542)	0.020	1.743(1.133–2.680)	0.011

^a^ Adjusted for age, education, smoking habits, alcohol consumption, physical activity, serum creatinine, fasting glucose, and plasma insulin;

^b^ Adjusted for the above + CKD and DM.

As shown in Table 4, VAI was significantly associated with both prehypertension and hypertension in females following adjustments for age, education, smoking habits, alcohol consumption, physical activity, serum creatinine, fasting glucose, and plasma insulin; the ORs for prehypertension and hypertension in the upper quartile of the VAI were 1.691 (95%CI 1.223–2.338), P = 0.001, and 1.688 (95%CI 1.220–2.334), P = 0.002, in females. Following further adjustments for the above confounders, CKD, and DM, the ORs for prehypertension and hypertension in the upper quartile of the VAI were 1.682 (95%CI 1.162–2.435), P = 0.006, and 1.657 (95%CI 1.141–2.406), P = 0.008, in females.

**Table 4:** The relationship between visceral adiposity index and blood pressure levels in females.

VAI	Prehypertension (Female)				Hypertension (Female)			
	Model one ^a^		Model two ^b^		Model one ^a^		Model two ^b^	
	OR(95% CI)	*P*	OR(95% CI)	*P*	OR(95% CI)	*P*	OR(95% CI)	*P*
1^st^ Quartile	Reference		Reference		Reference		Reference	
2^nd^ Quartile	1.321(0.974–1.792)	0.073	1.326(0.978–1.798)	0.070	1.039(0.710–1.519)	0.845	1.022(0.697–1.499)	0.911
3^rd^ Quartile	1.517(1.114–2.066)	0.008	1.516(1.113–2.064)	0.008	1.273(0.881–1.839)	0.199	1.294(0.894–1.873)	0.172
4^th^ Quartile	1.691(1.223–2.338)	0.001	1.688(1.220–2.334)	0.002	1.682(1.162–2.435)	0.006	1.657(1.141–2.406)	0.008

^a^ Adjusted for age, education, smoking habits, alcohol consumption, physical activity, serum creatinine, fasting glucose, and plasma insulin;

^b^ Adjusted for the above + CKD and DM.

## Discussion

Our research revealed that there was a significant relationship between the VAI and blood pressure in both men and women, a relationship independent of age, smoking habits, alcohol consumption, physical activity, education, Scr, serum glucose, plasma insulin, CKD and DM. Furthermore, the risk of prehypertension was higher in the upper quartile of the VAI compared with the lower quartile; the ORs were 1.533 (1.086–2.165), P = 0.015, in males, and 1.688 (95% CI 1.220–2.334), P = 0.002, in females. Our pilot study also performed a multivariate logistic regression using VAI values as continuous variables, the result showed that VAI score was independent risk factor of prehypertension and hypertension, the ORs were 0.747 (95%CI 2.583–0.957), P = 0.021, in prehypertension, and 0.561 (95%CI 0.418–0.752), P = 0.000, in hypertension, in males; the ORs were 1.185 (95%CI 1.024–1.371), P = 0.023, in prehypertension, and 1.239 (95CI 1.059–1.450), P = 0.008, in hypertension, in females. Our result was consistent with reports which revealed that VAI has significant correlation with hypertension [21, 22], and showed good predictive power regarding hypertension [23], however our current study revealed that VAI was also a good predictor regarding prehypertension.

There were reverse association between prevalence of current smoking as well as current alcohol consumption and VAI in males, and positive association between prevalence of inactive physical activity as well as chronic disease (CKD, DM and Hypertension) and VAI in males, according to Table 1, maybe it reflect the possible transition to healthy lifestyle in individuals who already have faced some chronic disease manifestations, however the underlying mechanism still need to be revealed in the future study. Generally speaking, well-educated middle aged individuals involve in more social activities such as business dinner or banquet, consuming more high calorie and high fat foods, and have no time to participate in outdoor activities, this may help to explain the reason why well-educated ladies have less physical activities according to Table 2. However, there was no directly evidence of reverse association between well-educated individuals and inactive physical activity, since it was not the same in males. Age, as well as serum creatinine, have positive correlation with VAI in females. Maybe the possible reason is that there were sizeable number of postmenopause women included in the upper quartile VAI of females. It’s a well known fact that age is closely related with serum creatinine. However, the relationship between serum creatinine and VAI need to be revealed in the future study.

The VAI is a sex-specific scoring system based on WC, BMI, TG and HDLC and is capable of providing information regarding visceral adipose tissue function and insulin sensitivity; it has recently been suggested as a surrogate of visceral adiposity. However, there is no ideal cut-off point at which to diagnose visceral adiposity. Another researcher used VAI tertiles (mild visceral adipose dysfunction, moderate visceral adipose dysfunction, and severe visceral adipose dysfunction) to determine an appropriate stratified cut-off point [24]. We used quartiles to both evaluate visceral adipose dysfunction and undertake a detailed analysis of the relationship between VAI and blood pressure. We also found that the 3rd quartile of the VAI correlated positively with prehypertension in both model one and model two among women; the ORs were 1.517 (95% CI 1.114–2.066), P = 0.008, and 1.516 (95% CI 1.113–2.064), P = 0.008; the 3rd quartile of the VAI correlated positively with hypertension in model two among men; the OR was 1.542 (95% CI 1.015–2.343), P = 0.042. However, the upper quartile of the VAI correlated positively with both prehypertension and hypertension in both model one and model two. Therefore, the upper quartile of the VAI may be used as a criterion with which to evaluate visceral adipose dysfunction.

Visceral adiposity is almost well-validated for prediction of metabolic syndrome [25–29], however sparse data about VAI and metabolic syndrome reported. Therefore, a multivariate logistic regression was also performed in order to check the relationship between VAI and metabolic syndrome. According to the diagnostic criteria of metabolic syndrome recommended by American Heart Association 2009, elevated blood pressure was defined as systolic≥130 and/or diastolic≥85 mm Hg, or antihypertensive drug treatment in a patient with a history of hypertension [30]. Our results revealed that VAI score was independent risk factor of metabolic syndrome, the ORs were 6.279 (95%CI 2.813–14.018), P = 0.000, in males, and 1.854 (95CI 1.324–2.595), P = 0.000, in females; if VAI values were used as continuous variables, the ORs were 1.771 (95CI 1.177–2.663), P = 0.006, in males, and 1.073 (95%CI 1.016–1.134), P = 0.011, in females. VAI, WC, and waist-height ratio (WHtR) were the best predictors of the individual components of the metabolic syndrome among Peruvian adults [31], more and more studies come to an agreement that VAI was a good marker of metabolic syndrome [32, 33].

VAI has a strong independent association with cardiovascular, OR = 2.45, 95%CI 1.52–3.95 [5]. VAI scores significantly increased in metabolically healthy obese individuals than metabolically healthy normal-weight individuals, and was an nontraditional risk factor of CVD [34]. VAI increase cardiometabolic risk in type 2 diabetes, and was significantly decreased after 12 month’s intervention with medicine [35]. VAI is predictive for cardiovascular events in prevalent hemodialysis patients [36] and polycystic ovary syndrome [21]. Increased adipokine production and proinflammatory activity caused by VAI, may served as accumulating evidence for identifying inflammation as a potential mechanism linking adipose tissue and cardiometabolic risk [37].

The VAI was significantly increased in prehypertension in our study, a scenario associated with an increased risk of CVD. Therefore, our study based on a cross-sectional epidemiological survey, maybe suitable for managing risk factors of prehypertension, and prevent the progression of normalcy and prehypertension to hypertension.

## Limitations

The VAI was established in Caucasian populations [5], its suitability for other populations needs to be further investigated, and each population and ethnic group should have their own VAI constants. However, one cross-sectional study have already validated its suitability in Chinese human [38], and our research revealed that the VAI correlates positively with BP. But there still remains much to develop a well-designed study to determine the mechanism underlying this relationship.

## Conclusions

VAI was associated with blood pressure and significantly increased in prehypertension.
